# Use of private vaccination services by infants in Brazilian municipalities: National Vaccine Coverage Survey 2020

**DOI:** 10.1590/S2237-96222024v33e20231203.especial2.en

**Published:** 2024-11-01

**Authors:** Ediane de Fátima Mance Burdinski, Maiara Sulzbach Denardin, Gisele Marins, Sandra Duran Otero, Ana Paula França, José Cássio de Moraes, Karin Regina Luhm, ICV 2020 Group, Adriana Ilha da Silva, Alberto Novaes Ramos, Ana Paula França, Andrea de Nazaré Marvão Oliveira, Antonio Fernando Boing, Carla Magda Allan Santos Domingues, Consuelo Silva de Oliveira, Ethel Leonor Noia Maciel, Ione Aquemi Guibu, Isabelle Ribeiro Barbosa Mirabal, Jaqueline Caracas Barbosa, Jaqueline Costa Lima, José Cássio de Moraes, Karin Regina Luhm, Karlla Antonieta Amorim Caetano, Luisa Helena de Oliveira Lima, Maria Bernadete de Cerqueira Antunes, Maria da Gloria Teixeira, Maria Denise de Castro Teixeira, Maria Fernanda de Sousa Oliveira Borges, Rejane Christine de Sousa Queiroz, Ricardo Queiroz Gurgel, Rita Barradas Barata, Roberta Nogueira Calandrini de Azevedo, Sandra Maria do Valle Leone de Oliveira, Sheila Araújo Teles, Silvana Granado Nogueira da Gama, Sotero Serrate Mengue, Taynãna César Simões, Valdir Nascimento, Wildo Navegantes de Araújo

**Affiliations:** 1 Universidade Federal do Paraná, Programa de Pós-graduação em Saúde Coletiva, Curitiba, PR, Brazil; 2 Universidade Federal do Paraná, Curso de Medicina, Curitiba, PR, Brazil; 3 Pesquisadora Autônoma, Curitiba, PR, Brazil; 4 Faculdade de Ciências Médicas da Santa Casa de São Paulo, Departamento de Saúde Coletiva, São Paulo, SP, Brazil; 5 Universidade Federal do Paraná, Departamento de Saúde Coletiva, Curitiba, PR, Brazil; Universidade Federal do Espírito Santo, Vitória, ES, Brazil; Universidade Federal do Ceará, Departamento de Saúde Comunitária, Fortaleza, CE, Brazil; Faculdade Ciências Médicas Santa Casa de São Paulo, São Paulo, SP, Brazil; Secretaria de Estado da Saúde do Amapá, Macapá, AP, Brazil; Universidade Federal de Santa Catarina, SC, Brazil; Organização Pan-Americana da Saúde, Brasília, DF, Brazil; Instituto Evandro Chagas, Belém, PA, Brazil; Faculdade de Ciências Médicas Santa Casa de São Paulo, Departamento de Saúde Coletiva, São Paulo, SP, Brazil; Universidade Federal do Rio Grande do Norte, Natal, RN, Brazil; Universidade Federal do Ceará, Departamento de Saúde Comunitária, Fortaleza, CE, Brazil; Universidade Federal de Mato Grosso, Cuiabá, MT, Brazil; Universidade Federal do Paraná, Curitiba, PR, Brazil; Universidade Federal de Goiás, Goiânia, GO, Brazil; Universidade Federal do Piauí, Teresina, PI, Brazil; Universidade de Pernambuco, Faculdade de Ciências Médicas, Recife, PE, Brazil; Instituto de Saúde Coletiva, Universidade Federal da Bahia, Salvador, BA, Brazil; Secretaria de Estado da Saúde de Alagoas, Maceió, AL, Brazil; Universidade Federal do Acre, Rio Branco, AC, Brazil; Universidade Federal do Maranhão, Departamento de Saúde Pública, São Luís, MA, Brazil; Universidade Federal de Sergipe, Aracaju, SE, Brazil; Secretaria Municipal de Saúde, Boa Vista, RR, Brazil; Fundação Oswaldo Cruz, Mato Grosso do Sul, Campo Grande, MS, Brazil; Fundação Oswaldo Cruz, Escola Nacional de Saúde Pública Sergio Arouca, Rio de Janeiro, RJ, Brazil; Universidade Federal do Rio Grande do Sul, Porto Alegre, RS, Brazil; Fundação Oswaldo Cruz, Instituto de Pesquisa René Rachou, Belo Horizonte, MG, Brazil; Secretaria de Desenvolvimento Ambiental de Rondônia, Porto Velho, RO, Brazil; Universidade de Brasília, Brasília, DF, Brazil

**Keywords:** Cobertura de servicios sanitarios privados, Cobertura de vacunación, Vacunas, Encuestas Epidemiológicas, Private Health Service Coverage, Vaccination Coverage, Vaccines, Epidemiological Survey

## Abstract

**Objective:**

To characterize the use of private services in infant vaccination and assess vaccination coverage according to the service used.

**Methods:**

**:** This was a national vaccination survey conducted in 2020 that estimated the use of private vaccination services and vaccination coverage among infants residing in state capitals and 12 inland municipalities.

**Results:**

**:** Of the 37,801 participants, 25.1% (95%CI 23.2;27.2) used private services at least once, with higher proportions in capitals, larger cities and in the South and Southeast regions. Socioeconomic and demographic differences were identified among families, based on the service used. The coverage for the set of vaccines administered up to 24 months was 60.3% (95%CI 58.6;62.0) in the public service and 59.5% (95%CI 55.9;63.0) in private services, and up-to-date vaccines, 10.3% (95%CI 9.1;11.6) and 9.4% (95%CI 7.4;11.8), respectively.

**Conclusion:**

The use of private services was frequent, with low coverage for the set of vaccines, regardless of the type of service used, especially for up-to-date vaccines.

## Study contributions

**Main results:**

The use of private vaccination services was observed in 25% of the municipalities studied, as well as low complete vaccination coverage in children up to 24 months old in both private and public services, especially for up-to-date vaccines.

**Implications for services:**

The increasing role of private vaccination services highlights the importance of coordination between immunization program managers at all levels and private vaccination services.

**Perspectives:**

Monitoring the vaccination status and conducting studies to understand the factors associated with incomplete and delayed vaccination are necessary. Such research can contribute to the adoption of new strategies that also include private vaccination services.

## RESUMEN

**Objetivo:**

Caracterizar el empleo de servicios privados en la vacunación de lactantes y evaluar la cobertura vacunal según el servicio utilizado.

**Método:**

Encuesta nacional de vacunación realizada en 2020, que estimó la utilización de servicios privados y cuberturas vacunales de lactantes residentes en capitales y 12 ciudades del interior.

**Resultados:**

De los 37.801 participantes, 9.287 (25,1% IC_95_% 23,2;27,2) utilizaron alguna vez servicios privados, con mayores proporciones en capitales, ciudades más grandes y regiones Sur y Sureste. Se identificaron diferencias socioeconómicas y demográficas en las familias, según servicio utilizado. La cobertura del conjunto de vacunas administradas hasta 24 meses fue 60,3% (IC_95_% 58,6;62,0) en servicios públicos y 59,5% (IC_95_% 55,9;63,0) en privados, y de vacunas al día 10,3% (IC_95_% 9,1;11,6) y 9,4% (IC_95_% 7,4;11,8) respectivamente.

**Conclusión:**

El uso de servicios privados fue frecuente, con baja cobertura para el conjunto de vacunas, independientemente del tipo de servicio utilizado, específicamente para vacunas al día.

## INTRODUCTION

Developed in 1973, the National Immunization Program (*Programa Nacional de Imunizações* - PNI) initially offered four vaccines.^1^ Currently, 19 vaccines are universally available and free of charge.^2^ The process of incorporating vaccines into PNI did not keep pace with the development of new vaccines in the 1980s, with a mismatch that dates back to the emergence of private vaccination services.^3^ Consequently, a complementary relationship between the public and private sectors was established, where private services, organized around clinics, medical offices and, more recently, pharmacies, consolidated their presence by offering vaccines not available in the PNI, or available only to specific age groups and populations through the Reference Centers for Special Immunobiologicals (*Centros de Referência para Imunobiológicos Especiais* - CRIE).^4-6^ In infant vaccination, this complementarity is evident as the private sector provides vaccines recommended by medical societies that are either not offered or offered in different formulations by the PNI.^7^

In many countries, the private sector plays a significant role in expanding vaccination.^8,9^ In low-income countries, these services provide access to routine vaccines, while in middle-income countries, they facilitate the adoption of new vaccines, before they are available through public services.^10^ The World Health Organization highlights the need for coordination between the public and private vaccination sectors,^11^ emphasizing the importance of monitoring private services to ensure the quality of vaccination.^12^

The growth in the participation of private vaccination services^8^ and the association between the vaccination service used and incomplete or delayed vaccination have been observed in different countries.^13^–^16^ In Brazil, vaccination coverage surveys have provided data on the services used for infant vaccination,^17^showing an increase in the use of private services, from 16% in 2007-2008^18^ to 23% in 2020,^17^ with higher vaccination completeness among infants who exclusively used public vaccination services in 2007-2008.^18^

Given the increasing role of private services in vaccination,^19^ This study aimed to characterize the use of private services in infant vaccination and assess vaccination coverage according to the service used.

## METHODS

### Study design

This was a population-based survey to assess vaccination coverage, conducted between September 2020 and March 2022, which is part of the National Vaccination Coverage Survey 2020 (*Inquérito Nacional de Cobertura Vacinal* - INCV 2020).^17^

### Setting, participants and study size

The study population was comprised of infants born alive in 2017 and 2018, whose mothers lived in private households located in urban areas of the 26 state capitals, in the Federal District and the municipalities of Campinas (São Paulo state), Caruaru (Pernambuco state), Imperatriz (Maranhão state), Joinville (Santa Catarina state), Londrina (Paraná state), Petrópolis (Rio de Janeiro state), Rio Grande (Rio Grande do Sul state), Rio Verde (Goiás state), Rondonópolis (Mato Grosso state), Sete Lagoas (Minas Gerais state), Sobral (Ceará state) and Vitória da Conquista (Bahia state).

The ICNV 2020 sampling plan^17^organized the census tracts into clusters according to socioeconomic strata. In order to define the sample size, the following parameters were taken into consideration: a design effect of 1.4; a hypothetical population of 1 million live births; an estimated vaccination coverage prevalence of 70%; an estimation error of 5%; and a 95% confidence interval – resulting in a sample of 452 infants per survey. Depending on the number of births recorded in the Live Birth Information System, one to four surveys were conducted in each city.^17^

### Variables

The study estimated the proportion of use of private vaccination services and compared the profiles of infants who used private services at least once with those who exclusively used public services and assessed coverage, according to the vaccination service used.

In order to determine the vaccination service used (private at least once or exclusively public) and estimate the number of infants who used private services at least once, the respondents’ answers to the question: *Has the child used any private vaccination services?* were taken into account. Given that different vaccine compositions are used by public and private services for protection against the same diseases, participants with records in the INCV 2020 database of receiving vaccines offered by private services, but not universally provided by the PNI, were also included to increase sensitivity: any dose of diphtheria, tetanus and acellular pertussis (DTPa) vaccine, DTPa vaccine, *Haemophilus influenzae* B and inactivated polio vaccine (IPV) or acellular pentavalent vaccine, DTPa, *Haemophilus influenzae* B, IPV and hepatitis B vaccine or acellular hexavalent vaccine, hepatitis A and B combined vaccine, meningococcal ACWY (MenACWY) vaccine, meningococcal B vaccine, second dose of the tetravalent measles, mumps, rubella and varicella (MMRV) vaccine and third dose of the human rotavirus vaccine. 

Other vaccines offered by private services, also offered by the PNI or in different formulations, were recorded in the database in a variable that combined various presentations, without differentiating whether the vaccine was administered in private or public services, making it impossible to evaluate these vaccines. For example, the MMRV vaccine, regardless of whether it was administered in public or private services, was included in the doses of the measles, mumps and rubella (MMR) vaccine or triple viral vaccine, chickenpox vaccine and the 13-valent pneumococcal conjugate vaccine (PCV13) was added to the 10-valent pneumococcal vaccine (PCV-10).

To estimate the proportion of the use of private vaccination services, the following formula was used:


$$\frac{\text{No. of infants who used private vaccination services at least once}}{\text{No. of infants in the sample}}\times 100$$

In order to compare the profile of infants who used private vaccination services at least once with those who have exclusively used public services, the following variables were considered:

characteristics of the infants:
sex (male and female);
birth order among siblings (first-born, second-born, third-born and fourth-born or later).

mother’s characteristics:
schooling (in years: ≤ 8, 9-12, 13-15, and ≥ 16);
number of living children (one, two, three and four, or more);
age at the child´s birth (in years: ≤ 20, 20-34, ≥ 35);
marital status (with or without a partner);
paid work (yes and no);
race/skin color (White, Black, mixed-race, Asian, Indigenous).

family and household characteristics:
family consumption level (A, B, C and D – according to the classification of the Brazilian Association of Research Companies, with A being the highest level and D being the lowest level);
socioeconomic stratum of the area of residence (A – high, B – medium-high, C – medium-low and D – low); monthly household income (in BRL: ≤ 1,000, 1,001-3,000, 3,001-8,000 and ≥ 8,001).

The socioeconomic strata of the area of residence were classified based on the income and literacy of the head of household.^17^

Coverage was assessed for each vaccine recommended by the PNI up to 24 months old, taking into consideration the dose related to the complete schedule or booster, and for the set of these vaccines (complete coverage). Up to 12 months old, the following were considered: first dose of Bacillus Calmette -Guérin (BCG) or tuberculosis vaccine and hepatitis B vaccine; second dose of PCV10, human rotavirus and meningococcal C conjugate (MenC) vaccine; and third dose of the pentavalent vaccine and IPV vaccine. The yellow fever vaccine was not included, as it was not part of the routine schedule in all municipalities included in the study during the period analyzed. From 12 to 24 months old, the following were considered: first booster of PCV10, MenC, oral poliovirus vaccine (OPV) and diphtheria, tetanus and pertussis (DTP) vaccine; first dose of the hepatitis A vaccine and chickenpox vaccine; and second dose of MMR.

Vaccination coverage was calculated for vaccines administered (considering all administered doses recorded in the vaccination booklet) and up-to-date vaccine (considering only doses administered within 30 days after the date scheduled by the PNI), using the following formulas:


$$\text{Coverage for administered vaccines}=\frac{\text{No. of infants with the vaccine administered}}{\text{No. of infants in the sample}}\times 100$$

$$\text{Coverage for up-to-date vaccines}=\frac{\text{No. of infants with up−to−date vaccines}}{\text{No. of infants in the sample}}\times 100$$

$$\text{Complete coverarage for administered vaccines}=\frac{\text{No. of infants with all vaccines scheduled up to 24 months administered}}{\text{No. of infants in the sample}}\times 100$$

$$\text{Complete coverage for up-to-date vaccines}=\frac{\text{No. of infants with all vaccines scheduled up to 24 months administered up to date}}{\text{No. of infants in the sample}}\times 100$$

The proportion of use of private vaccination services and complete coverage were described by:

municipality:
grouped according to interiority (inland cities and capitals);
grouped according to population size (per/thousand inhabitants), based on the classification of the Brazilian Institute of Geography and Statistics (150 – 900,000 inhabitants and > 900,000 inhabitants);

regions (Midwest, Northeast, North, South and Southeast).

The proportion of use of private vaccination services was also described for each municipality. Coverage according to the vaccines was presented for the set of municipalities taking part in the survey.

### Data sources

The data were obtained through interviews with the infants’ guardians, as well as by transcribing information about vaccines administered up to 24 months old from photographs of the infants’ vaccination booklets.^17^

### Statistical methods

The proportions of use of private vaccination services, vaccination coverage and 95% confidence intervals were calculated using the Stata software, version 17. Pearson’s chi-square test was used to test for statistical differences. A p-value < 0.05 was considered statistically significant. To correct for potential distortions in the sample distribution and allow for unbiased estimates, the survey analysis module was used, taking into account the socioeconomic stratum of the area of residence, calibration weights and cluster. Missing data were tabulated together with “Don’t know” responses; both were included in the analyses.

#### Ethical aspects

The survey was approved by the Research Ethics Committees of the Instituto de Saúde Coletiva da Universidade Federal da Bahia (opinion 3.366.818, on 6/4/2019, Certificate of Submission of Ethical Appraisal [CAAE] 4306919.5.0000.5030); and the Irmandade da Santa Casa de São Paulo (opinion 4,380,019, 11/4/2020, CAAE 39412020.0.0000.5479). The informed consent form was signed by the infants’ guardians.^17^

## RESULTS

Of the expected sample of 39,776 infants, 37,801 were included in the survey. The losses accounted for 6%, varying across municipalities and strata, resulting from refusals, the inability to conduct the interview after three attempts and the failure to locate the expected number of children after an active search.

Among the infants taking part in the survey, 8,536 guardians reported using private services. Analysis of the vaccine records identified an additional 751 participants, totaling 9,287 (25.1%; 95%CI 23.2;27.2) infants who had used private vaccination services at least once.

Differences were identified in the socioeconomic and demographic profile of families, based on the service used, except for the sex of the infant. When comparing infants who had used private services at least once with those who had only used public services, it could be seen a higher proportion of first-born children (61.8% and 43.2%, respectively), whose mothers had ≥ 16 years of education (75.9% and 16.8%, respectively), aged 35 years or older (70% and 32.8%, respectively), engaged in paid work (72.8% and 47.0%, respectively), and who self-identified as White (69.9% and 36.2%, respectively). Higher proportions of families classified in socioeconomic levels A and B, considered to have higher consumption levels (66.4% and 12.1%, respectively) were also observed. The low proportion of infants who used private services among mothers with ≤ 8 years of education (1.4% and 20.0%, respectively) and whose families reported a monthly income ≤ BRL 1,000 (6.7% and 32.9%, respectively), when comparing the use of vaccination services, stands out. (Table 1).

**Table 1:** Infant, maternal and family characteristics, according to the use of private vaccination services^a^ in Brazilian municipalities, National Vaccine Coverage Survey, 2020 (n = 37,801)

Use of private vaccination services	Yes (9,287)			No (28,514)			p-value
	**N**	**%**	**95%CI**	**n**	**%**	**95%CI**	
Characteristics of infants							
Sex							0.230
Masculine	4,769	49.4	46.3;52.5	14,638	51.6	49.9;53.2	
Feminine	4,518	50.6	47.5;53.7	13,876	48.4	46.8;50.1	
Birth order							< 0.001
First-born	5,589	61.8	58.6;65.0	12,670	43.0	41.2;44.9	
Second-born	2,977	29.7	26.7;32.8	9,113	31.7	30.3;33.1	
third-born	553	6.5	4.9;8.5	4,155	15.0	13.9;16.1	
Fourtd-born or later	157	2.0	1.2;3.1	2,553	10.2	9.0;11.5	
**Maternal characteristics**							
**Schooling (in years of study)**							< 0.001
≤ 8	115	1.4	0.9;2.2	3,165	20.0	9.9;12.1	
9-12	231	3.4	2.4;4.9	5,263	19.6	18.0;21.2	
13-15	1,478	17.6	15.2;20.1	13,859	49.3	47.4;51.1	
≥ 16	7,311	75.9	72.7;78.6	5,326	16.8	15.3;18.4	
Don’t know/didn’t answer	152	1.7	1.1;2.5	901	3.3	2.7;3.9	
**Number of living children**							< 0.001
One	4,317	49.1	45.6;52.7	10,024	33.3	31.7;35.0	
Two	3,965	39.2	35.7;42.8	10,062	35.7	33.9;37.4	
three	776	9.1	7.2;11.5	5,035	17.7	16.4;19.1	
Four or more	220	2.5	1.7;3.6	3,594	10.5	9.4;11.8	
**Age at the child’s birth (in years)**							< 0.001
≤ 20	30	3.6	0.2;0.6	832	3.1	2.5;3.7	
20-34	2,920	29.7	26.7;32.8	18,797	64.1	62.5;65.8	
≥ 35	6,302	70.0	66.8;72.9	8,729	32.8	31.1;34.4	
Don’t know/didn’t answer	26	4.5	0.1;1.5	156	0.5	0.3;0.7	
**Marital status**							< 0.001
With partner	8,266	88.4	86.2;90.3	19,975	69.3	67.7;70.8	
Without a partner	856	9.7	8.0;11.6	7,543	27.3	25.8;28.8	
Don’t know/didn’t answer	165	1.9	1.2;2.9	996	3.4	2.9;4.0	
**Paid work**							< 0.001
Yes	6,882	72.8	69.7;75.8	13,564	47.0	45.4;48.6	
No	2,259	25.7	22.8;28.7	14,161	50.2	48.6;51.7	
Don’t know/didn’t answer	146	1.5	0.9;2.3	789	2.8	2.3;3.4	
**Race/skin color**							< 0.001
White	6,365	69.9	66.3;73.3	8,862	36.2	34.0;38.5	
Black	375	6.0	4.3;8.4	3,880	14.7	13.5;16.0	
Mixed-race	2,252	20.5	19;23.1	14,607	44.7	42.8;46.7	
Asian	129	2.1	1.3;3.3	239	1.2	0.7;1.9	
Indigenous	10	0.0	0.0;0.1	115	0.4	0.2;0.5	
Don’t know/didn’t answer	156	1.4	0.9;2.2	811	0.3	2.2;3.3	
**Family characteristics**							
**Socioeconomic stratum of the area of residence**							< 0.001
A	3,642	27.2	23.0;31.9	4,691	6.7	5.6;7.9	
B	2,897	22.0	18.4;26.0	6,521	8.9	7.7;10.3	
C	2003	25.8	21.9;30.0	7,989	19.17	17.3;21.1	
D	745	24.9	20.8;29.5	9,313	65.2	62.3;68.0	
**Household consumption level**							< 0.001
A	1,645	14.4	11.9;17.4	290	0.8	0.6;1.2	
B	5,135	52.0	48.0;55.7	3,866	11.3	10.0;12.6	
C	1,747	22.4	19.3;25.7	9,868	37.6	35.6;39.6	
D	488	7.8	6.1;9.9	13,520	46.8	44.7;49.0	
Don’t know/didn’t answer	272	3.4	2.4;4.9	970	3.5	2.9;4.1	
**Monthly household income (in BRL)**							< 0.001
≤ 1,000	359	6.7	5.1;8.6	8,327	32.9	30.8;35.1	
1,000-3,000	1,115	13.6	11.1;16.6	11,551	40.9	38.8;43.1	
3,000-8,000	2,718	25.2	22.0;28.7	4,741	13.0	11.7;14.4	
≥ 8,001	3,646	29.6	26.1;33.4	974	2.3	1.9;2.9	
Don’t know/didn’t answer	1,449	24.8	20.8;29.4	2,921	10.7	10.0;12.6	

a) Yes: participants who have used private vaccination services at least once; No: participants who exclusively used public vaccination services.

**Figure 1:**
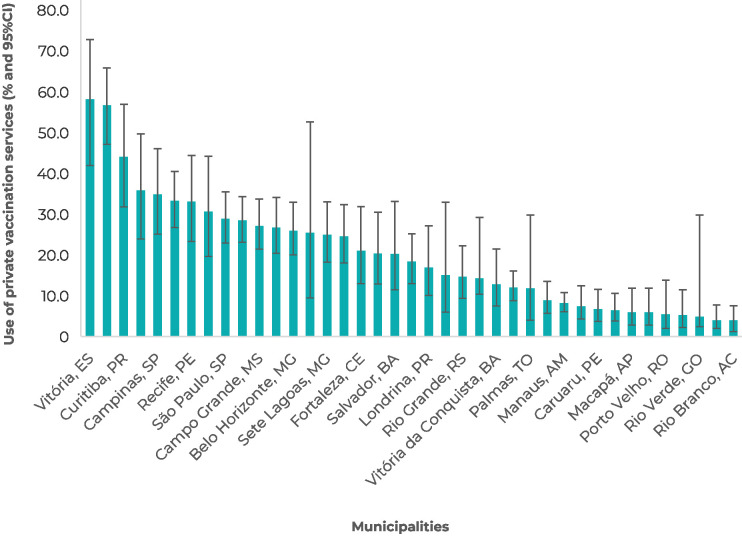
Use of private vaccination services^a^ by municipalities, National Vaccine Coverage Survey, 2020 (n = 37,801)

**Figure 2:**
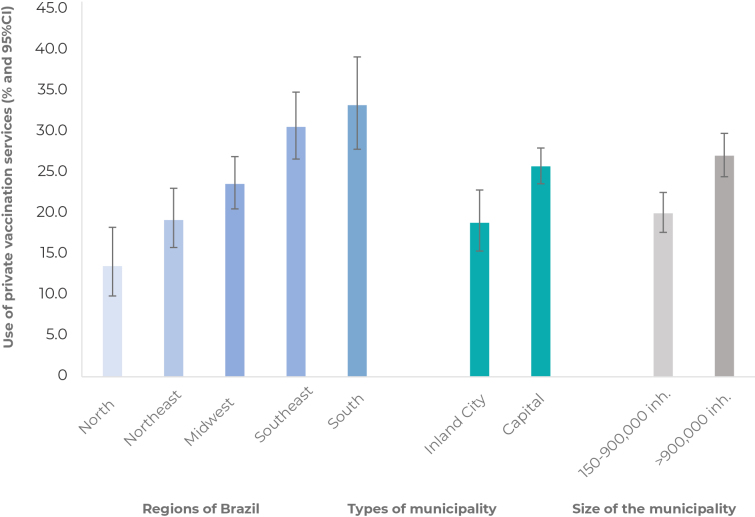
Use of private vaccination services^a^ according to Brazilian regions, interiority and population size of the municipality, National Vaccine Coverage Survey, 2020 (n = 37,801)

It could be seen territorial differences in the use of private vaccination services, ranging from 3.9% (95%CI 1.2;7.6), in Rio Branco, to 58.4% (95%CI 42.1;73 ,1), in Vitória (Figure 1), being higher in capitals (p = 0.004) and in municipalities of level 5 (p < 0.001). Proportions higher than 20% were found in 14 of the 26 capitals, in Brasília, and in three inland cities in the Southeast region, exceeding 50% in Vitória and Florianópolis. There was higher use of private vaccination services in the South and Southeast regions (33.2%; 95%CI 27.8;39.1 and 30.5%; 95%CI 26.6;34.8, respectively), and lower use in the North and Northeast regions (13.5%; 95%CI 9.9;18.3 and 19.2%; 95%CI 15.8;23.0, respectively) (p < 0.001) (Figure 2).

Taking into consideration the municipalities as whole, no differences were found in complete coverage up to 24 months old among infants who exclusively used public services or those who had used private services at least once. Complete coverage according to vaccines administered was 60.3% and 59.5%, respectively (95%CI 58.6;62.0, 95%CI 55.9;63.0, p = 0.704), and up-to-date vaccinations was 10.3% and 9.4% (95%CI 9.1;11.6, 95%CI 7.4;11.8, p = 0.473). Significant differences (p = 0.034) were observed only in the North region for up-to-date vaccinations, with coverage of 3.0% (95%CI 2.3;4.1) for the public service and 0.7% (95%CI 0.2;3.0) for private services (Table 2).

**Table 2:** Vaccination coverage for administered and up-to-date vaccines among the set of vaccines recommended up to 24 months old according to the use of private health services,^a^ based on the characteristics of the municipalities and regions of the country, National Vaccine Coverage Survey, Brazil (n = 37,801)

**Vaccination coverage**	**Administered vaccines**			**Up-to-date vaccines**		
	**Private (%)**	**Public (%)**	**p-value**	**Private (%)**	**Public (%)**	**p-value**
Brazil						
Total	59.5	60.3	0.704	9.4	10.3	0.473
**Municipality type**						
Inland city	64.6	61.7	0.538	10.6	10.9	0.885
Capital	59.2	60.1	0.656	9.3	10.2	0.492
**Municipality size (per/thousand inhabitants)**						
150-900	54.6	57.8	0.211	7.1	6.5	0.633
>900	60.8	61.3	0.859	10.0	11.8	0.274
**Regions**						
North	53.8	55.7	0.762	0.7	3.0	0.034
Northeast	50.7	58.6	0.122	5.8	9.0	0.098
Midwest	61.3	63.5	0.539	10.0	10.7	0.694
Southeast	61.1	60.3	0.824	11.6	13.6	0.410
South	67.1	68.4	0.753	9.0	10.0	0.675

a) Private: participants who had used private vaccination services at least once; Public: participants who exclusively used public vaccination services.

When evaluating coverage according to vaccines administered, greater coverage of the first dose of chickenpox was observed among infants who used private services. Among vaccines recommended up to 12 months old, up-to-date coverage of the second dose of PCV-10, the human rotavirus vaccine and MenC and the third dose of pentavalent vaccine and IPV was higher among infants who used private services. For vaccines recommended between 12 and 24 months old, up-to-date coverage of the first booster of OPV and the second dose of MMR was higher among infants who used public services; on the other hand, up-to-date coverage of the first dose of MMR, the hepatitis A and chickenpox vaccines and the first booster of PCV-10 was higher among infants who used private services (Table 3).

**Table 3:** Vaccination coverage for administered and up-to-date vaccines, by vaccines recommended up to 12 months old and from 12 to 24 months old, according to the type of service used for vaccination, ^a^ **National Vaccine Coverage Survey, Brazil, 2020 (n = 37,801)**

**Vaccine**	**Administered vaccines**			**Up-to-date vaccines**		
	**Public**	**Private**	**p-value**	**Public**	**Private**	**p-value**
	**% (95%CI)**	**% (95%CI)**		**% (95%CI)**	**% (95%CI)**	
**Up to 12 months old**						
First dose of tuberculosis vaccine	89.6 (87.3;91.5)	89.8 (88.5;91.0)	0.839	82.3 (79.3;85.0)	84 (82.6;85.4)	0.293
First dose of hepatitis B vaccine	89.3 (87.0;91.2)	88.6 (87.2;89.8)	0.537	86 (83.5;88.3)	85.5 (84.1;86.9)	0.756
Second dose of 10 valent pneumococcal vaccine	91.1 (89.0;92.8)	90.2 (89.0;91.3)	0.407	78.7 (78.8;81.3)	66.3 (64.4;68.0)	< 0.001
Second dose of human rotavirus vaccine	83.6 (80.7;86.1)	81.7 (80.3;83.1)	0.248	72.8 (69.2;76.0)	64.5 (62.7;66.4)	< 0.001
Second dose of meningococcal C vaccine	89.8 (87.7;91.5)	89.3 (88.1;90.4)	0.663	70.9 (67.8;73.8)	58.7 (56.7;60.7)	< 0.001
third dose of the pentavalent vaccine	86.7 (84.0;89.0)	88.6 (87.3;89.8)	0.148	66.1 (62.8;69.3)	53 (50.8;55.2)	< 0.001
third dose of inactivated polio vaccine	86.3 (83.6;88.6)	88.6 (87.3;89.8)	0.084	66.8 (63.3;70.0)	55.5 (53.5;57.5)	< 0.001
**12-24 months old**						
First booster of 10 valent pneumococcal vaccine	86.1 (83.4;88.4)	84.7 (83.3;85.9)	0.309	65.7 (62.6;68.7)	51.5 (49.7;53.3)	< 0.001
First booster of meningococcal C vaccine	84.0 (81.4;86.3)	81.6 (80.2;82.8)	0.108	48.0 (45.1;51.3)	47.4 (45.5;49.3)	0.229
First dose of hepatitis A vaccine	89.6 (87.4;91.4)	87.7 (86.4;88.9)	0.112	56.5 (53.3;59.7)	49.3 (47.7;50.9)	< 0.001
Second dose of MMR (measles, mumps and rubella) vaccine	84.3 (81.2;87.0)	81.5 (80.1;82.9)	0.103	39.8 (36.3;43.4)	42.7 (41.0;44.0)	0.019
First booster of oral poliovirus vaccine	84.4 (81.8;86.8)	86.9 (85.6;87.9)	0.078	39.5 (36.5;42.6)	44.3 (42.5;46.1)	0.039
First booster of DTP (diphtheria, tetanus and pertussis) vaccine	82.9 (80.2;85.3)	84.7 (83.4;85.9)	0.218	37.1(33.9;40.4)	37.2 (35.4;39.0)	0.427
First dose of chickenpox vaccine	89.3 (87.1;91.1)	86.3 (84.9;87.6)	0.019	57.2 (54.0;60.3)	44.1 (42.4;45.9)	< 0.001

a) Private: participants who had used private vaccination services at least once; Public: participants who exclusively used public vaccination services.

## DISCUSSION

This study addresses a topic that has been little discussed, which made it possible to characterize the use of private services for infant vaccinations in the country. One in every four infants taking part in INCV 2020 has used private services for vaccination at least once, with higher proportions observed in capitals, larger cities and the South and Southeast regions. Low complete coverage up to 24 months old was found, especially for vaccines administered on schedule, with no statistically significant differences between infants who used public and private vaccination services.

Comparing the current survey data from the capitals with those from 2007-2008 survey, there was a 60% increase in the use of private vaccination services^18^ and the number of capitals where the use of private services exceeded 20%, increased from three to 15.^20^ The increased participation of private services in childhood immunization has also been observed in other countries, such as India and Sri Lanka.^8,9^

Demographic differences in the use of private vaccination services may be associated with the level of local socioeconomic development and the greater presence of private services in these regions, making it easier for the population to access these services. It is worth highlighting that INCV 2020 included only capitals and inland municipalities with more than 100,000 inhabitants, which may partly explain the high proportions of use of private vaccination services observed. Similarly, previous studies estimated a 37% use of these services in the capital of Sri Lanka,^9^and 35% in the capital of Argentina^14^ contrasting with the 9% found in a study conducted in municipalities with more than 20,000 inhabitants across all regions of Argentina.^21^

Factors contributing to the significant participation of private services in infant vaccination are: the introduction of new vaccines not immediately incorporated into the PNI; shortage of some immunobiological agents in the Brazilian National Health System (Sistema Único de Saúde – SUS) vaccination rooms;^22^ the convenience of private services, including extended hours,^10,13^ proximity to residence in areas of higher socioeconomic stratum^10^ and, more recently, the administration of vaccines in pharmacies.^5^

In Brazil, public and private healthcare services coexist, sharing users.^23^ Given that immunization is not part of the mandatory procedures defined by the Brazilian National Supplementary Health Agency, private health insurance and plans generally do not cover vaccines and their insured persons routinely use public services for vaccination^24^ or pay directly for vaccines in private services. Higher proportions of use of private vaccination services by children whose parents had health insurance plans have been described,^21^ which may indicate that health plans facilitate access to vaccines not universally offered by the PNI.

Regarding socioeconomic profile, the greater use of private vaccination services by infants from families with higher consumption level and household income, and whose mothers were engaged in paid work, points to financial availability as an important factor in access to these services. Socioeconomic stratum, income and social class were associated with greater use of private services in India and Sri Lanka.^8,9^

If, on the one hand, the highest use of private services by infants whose mothers had a higher level of education may indicate greater knowledge about vaccines available in private services, on the other hand it may represent a confounding factor for variables such as income. Mothers who have a partner is another characteristic that could confound income. Similar to our findings, in the United States, children of parents who did not have higher education and who were single used public vaccination services more often.^16^ On the other hand, a study that evaluated vaccination expenditures in the adult population with diabetes, in São Paulo, did not identify differences in the use of public services according to income, education level and marital status.^25^

The birth order of the participant and the number of living children influenced the use of private vaccination services, with higher use for firstborns. These finds were also found in Sri Lanka.^9^ The inverse relationship between the number of children and the use of private services, especially from the third-born child onwards, may indicate a shift towards the use of public services due to economic pressure.

Taking into consideration the assessment of complete coverage up to 24 months old, infants who used public and private vaccination services showed similar vaccination coverage. Corroborating our results, Agampodi et al. did not find differences in coverage according to service type.^9^ Contrary to our findings, the 2007-2008 survey showed a higher likelihood of being fully vaccinated at 18 months old among infants who were exclusively vaccinated in public services.^18^ In an opposite trend, studies conducted in Argentina and the United States associated complete vaccination with exclusive use of private vaccination services and the concomitant use of both services.^14,15^ Furthermore, data from a national immunization survey in the United States, which assessed coverage at 24 months old, showed higher coverage among children with private health insurance.^26^

 With regard to the evaluation according to vaccines administered, coverage for the first dose of chickenpox was higher among infants who used private services. A possible explanation for this situation is the difference in the recommended age for this vaccine in the schedules adopted by the services, with it being recommended at 12 months old in private services and at 15 months old in public services.^2,7^

For more than 50% of the vaccines evaluated, higher up-to-date coverage was observed among infants who used private services. However, when assessing complete coverage, no differences were identified according to the type of vaccination service used, with very low coverage in both. A study in Singapore showed similar data regarding delays in receiving one or more doses of vaccine, but differing results when analyzing the service used, with delayed vaccination being more frequent in children vaccinated in the private sector or by multiple providers.^13^ Similarly, Simpson et al. identified better proportions of up-to-date vaccinations among children vaccinated in public services, when compared to those vaccinated in private services.^16^

The limitations of this study include the potential for bias in classifying participants according to the use of private services. Information from guardians regarding the use of a private service for vaccination may be inaccurate, as the person who answered the questionnaire was not always the family member who took the child for vaccination. Another limitation was the fact that the service used for each dose of vaccine was not transcribed from the vaccination booklets to the database, making it impossible to identify all doses administered by private services, especially for vaccines available in the PNI, which may have underestimated the use of these services. This situation also did not allow for the identification of infants who received all doses in private services. In order to identify the maximum number of infants who had used private services at least once, participants with records in the database of the administration of some vaccines offered by these services, which are not universally available in the PNI, were included. However, it is worth mentioning that these vaccines could have been administered at CRIE,^6^ which could, on the other hand, overestimate the use of private services. 

The frequency of use of private services observed highlights the importance of coordination between public and private vaccination services, to ensure the quality of vaccine administration and the accurate recording of doses administered in the PNI information system.^8,12,27^ Although it is mandatory to record individual data regarding vaccines administered by private services in the information system,^28,29^ there are no national strategies for monitoring private services.

Exclusive access to some vaccines by those who can use private vaccination services contradicts the principles of equity and universality of the SUS.^4,30^ However, it is observed that the PNI, with a decentralized service structure, throughout the SUS primary care network, has made a significant contribution to reducing social and regional inequalities, enabling access to vaccination for all Brazilians, in all locations.^1^ The similarity in complete coverage according to vaccines administered and up-to-date vaccines among infants who use public and private services reinforces the extensive reach of the PNI across Brazilian territory.

The unmet vaccination coverage goals and the administration of vaccines outside the recommended period, regardless of the service used, indicate the need to implement actions to address this situation. Detailed monitoring of the vaccination status, including the evaluation of the record of doses administered by private services in the PNI information system and understanding the factors associated with incomplete and delayed vaccination, can guide the adoption of new strategies aimed at recovering high coverage and ensuring up-to-date vaccination.
