# Supplementary material for: Use of private vaccination services by infants in Brazilian municipalities: National Vaccine Coverage Survey 2020
Source: Epidemiol Serv Saude. 2024 Nov 1;33(spe2):e20231203. doi: 10.1590/S2237-96222024v33e20231203.especial2.en (PMC11537176; doi:10.1590/S2237-96222024v33e20231203.especial2.en)
Supplement: Translation

# Translation: Utilização de serviços privados de vacinação por lactentes em municípios brasileiros: Inquérito Nacional de Cobertura Vacinal 2020

**DOI:** 10.1590/S2237-96222024v33e20231203.especial2.pt

**Authors:** Ediane de Fátima Mance Burdinski, Maiara Sulzbach Denardin, Gisele Marins, Sandra Duran Otero, Ana Paula França, José Cássio de Moraes, Karin Regina Luhm, Grupo ICV 2020, Adriana Ilha da Silva, Alberto Novaes Ramos, Ana Paula França, Andrea de Nazaré Marvão Oliveira, Antonio Fernando Boing, Carla Magda Allan Santos Domingues, Consuelo Silva de Oliveira, Ethel Leonor Noia Maciel, Ione Aquemi Guibu, Isabelle Ribeiro Barbosa Mirabal, Jaqueline Caracas Barbosa, Jaqueline Costa Lima, José Cássio de Moraes, Karin Regina Luhm, Karlla Antonieta Amorim Caetano, Luisa Helena de Oliveira Lima, Maria Bernadete de Cerqueira Antunes, Maria da Gloria Teixeira, Maria Denise de Castro Teixeira, Maria Fernanda de Sousa Oliveira Borges, Rejane Christine de Sousa Queiroz, Ricardo Queiroz Gurgel, Rita Barradas Barata, Roberta Nogueira Calandrini de Azevedo, Sandra Maria do Valle Leone de Oliveira, Sheila Araújo Teles, Silvana Granado Nogueira da Gama, Sotero Serrate Mengue, Taynãna César Simões, Valdir Nascimento, Wildo Navegantes de Araújo

**Affiliations:** 1 Universidade Federal do Paraná, Programa de Pós-graduação em Saúde Coletiva, Curitiba, PR, Brasil; 2 Universidade Federal do Paraná, Curso de Medicina, Curitiba, PR, Brasil; 3 Pesquisadora Autônoma, Curitiba, PR, Brasil; 4 Faculdade de Ciências Médicas da Santa Casa de São Paulo, Departamento de Saúde Coletiva, São Paulo, SP, Brasil; 5 Universidade Federal do Paraná, Departamento de Saúde Coletiva, Curitiba, PR, Brasil; Universidade Federal do Espírito Santo, Vitória, ES, Brasil; Universidade Federal do Ceará, Departamento de Saúde Comunitária, Fortaleza, CE, Brasil; Faculdade Ciências Médicas Santa Casa de São Paulo, São Paulo, SP, Brasil; Secretaria de Estado da Saúde do Amapá, Macapá, AP, Brasil; Universidade Federal de Santa Catarina, SC, Brasil; Organização Pan-Americana da Saúde, Brasília, DF, Brasil; Instituto Evandro Chagas, Belém, PA, Brasil; Faculdade de Ciências Médicas Santa Casa de São Paulo, Departamento de Saúde Coletiva, São Paulo, SP, Brasil; Universidade Federal do Rio Grande do Norte, Natal, RN, Brasil; Universidade Federal do Ceará, Departamento de Saúde Comunitária, Fortaleza, CE, Brasil; Universidade Federal de Mato Grosso, Cuiabá, MT, Brasil; Universidade Federal do Paraná, Curitiba, PR, Brasil; Universidade Federal de Goiás, Goiânia, GO, Brasil; Universidade Federal do Piauí, Teresina, PI, Brasil; Universidade de Pernambuco, Faculdade de Ciências Médicas, Recife, PE, Brasil; Instituto de Saúde Coletiva, Universidade Federal da Bahia, Salvador, BA, Brasil; Secretaria de Estado da Saúde de Alagoas, Maceió, AL, Brasil; Universidade Federal do Acre, Rio Branco, AC, Brasil; Universidade Federal do Maranhão, Departamento de Saúde Pública, São Luís, MA, Brasil; Universidade Federal de Sergipe, Aracaju, SE, Brasil; Secretaria Municipal de Saúde, Boa Vista, RR, Brasil; Fundação Oswaldo Cruz, Mato Grosso do Sul, Campo Grande, MS, Brasil; Fundação Oswaldo Cruz, Escola Nacional de Saúde Pública Sergio Arouca, Rio de Janeiro, RJ, Brasil; Universidade Federal do Rio Grande do Sul, Porto Alegre, RS, Brasil; Fundação Oswaldo Cruz, Instituto de Pesquisa René Rachou, Belo Horizonte, MG, Brasil; Secretaria de Desenvolvimento Ambiental de Rondônia, Porto Velho, RO, Brasil; Universidade de Brasília, Brasília, DF, Brasil

**Keywords:** Cobertura de Serviços Privados de Saúde, Cobertura Vacinal, Vacinas, Inquérito Epidemiológico

## Abstract

**Objetivo:**

Caracterizar a utilização dos serviços privados na vacinação de lactentes e avaliar as coberturas vacinais segundo serviço utilizado.

**Métodos:**

Inquérito nacional de vacinação realizado em 2020 que estimou a utilização de serviços privados de vacinação e as coberturas vacinais de lactentes residentes nas capitais dos estados, no Distrito Federal e em 12 municípios do interior.

**Resultados:**

Dos 37.801 participantes, 25,1% (IC_95_% 23,2;27,2) utilizaram alguma vez serviços privados, com maiores proporções em capitais, cidades de maior porte e regiões Sul e Sudeste. Identificaram-se diferenças socioeconômicas e demográficas nas famílias, segundo o serviço utilizado. A cobertura para o conjunto de vacinas aplicadas até 24 meses foi de 60,3% (IC_95_% 58,6;62,0) no serviço público e de 59,5% (IC_95_% 55,9;63,0) no privado, e das vacinas em dia, de 10,3% (IC_95_% 9,1;11,6) e 9,4% (IC_95_% 7,4;11,8), respectivamente.

**Conclusão:**

A utilização dos serviços privados foi frequente, com baixas coberturas para o conjunto de vacinas, independentemente do tipo de serviço utilizado, especialmente para vacinas em dia.

## Contribuições do estudo

**Principais resultados:**

A utilização dos serviços privados de vacinação foi observada em 25% dos municípios estudados, assim como uma baixa cobertura vacinal completa em crianças até 24 meses nos serviços privados e públicos, especialmente para as vacinas em dia.

**Implicações para os serviços:**

A crescente participação dos serviços privados na vacinação aponta a importância da articulação entre os gestores dos programas de imunização de todas as instâncias e serviços privados de vacinação.

**Perspectivas:**

O monitoramento da situação vacinal e estudos para o conhecimento dos fatores associados à incompletude e ao atraso vacinal são necessários. Tal investigação pode contribuir para a adoção de novas estratégias que incluam também os serviços privados de vacinação.

## INTRODUÇÃO

Formulado em 1973, o Programa Nacional de Imunizações (PNI) ofertava quatro vacinas.^1^ Atualmente disponibiliza 19 vacinas de maneira universal e gratuita.^2^ O processo de incorporação de vacinas ao PNI não ocorreu na mesma velocidade que o desenvolvimento de novas vacinas na década de 1980, com descompasso que remontou à origem dos serviços privados de vacinação.^3^ Estabeleceu-se, assim, relação de complementariedade entre os segmentos público e privado, na qual os serviços privados, organizados em torno de clínicas, consultórios e, mais recentemente, em farmácias, se consolidaram por ofertarem vacinas não disponíveis no PNI, ou disponíveis apenas para faixas etárias e grupos específicos através dos Centros de Referência para Imunobiológicos Especiais (CRIE).^4^–^6^ Na vacinação de lactentes, a complementariedade se faz presente pela oferta, no segmento privado, de vacinas indicadas por sociedades médicas, não ofertadas ou com apresentação diferente das ofertadas pelo PNI.^7^

Em muitos países, o setor privado desempenha papel importante na ampliação da vacinação.^8,9^ Nos países de baixa renda, tais serviços possibilitam o acesso a vacinas básicas e, nos de média renda, facilitam a adoção de novas vacinas, antes da disponibilização delas pelos serviços públicos.^10^ A Organização Mundial da Saúde aponta a necessidade de coordenação entre os setores público e privado de vacinação,^11^ sendo essencial o monitoramento dos serviços privados para se garantir uma vacinação de qualidade.^12^

O crescimento da participação dos serviços privados de vacinação^8^ e a associação do serviço de vacinação utilizado com a incompletude ou atraso vacinal vêm sendo observados em diferentes países.^13^–^16^ No Brasil, inquéritos de cobertura vacinal têm permitido a obtenção de dados referentes aos serviços utilizados para vacinação de lactentes,^17^ evidenciando um aumento da utilização de serviços privados, que passou de 16%, em 2007-2008^18^, para 23%, em 2020,^17^ e maior completude vacinal entre lactentes que utilizavam exclusivamente serviços públicos de vacinação em 2007-2008.^18^

Considerando o crescimento da participação dos serviços privados na vacinação,^19^ o presente estudo buscou caracterizar a utilização de serviços privados na vacinação de lactentes e avaliar as coberturas vacinais segundo o serviço utilizado.

## MÉTODOS

### Delineamento

Trata-se de inquérito de base populacional para avaliação da cobertura vacinal, realizado entre setembro de 2020 e março de 2022, que integra o Inquérito Nacional de Cobertura Vacinal 2020 (INCV 2020).^17^

### Contexto, participantes e tamanho do estudo

A população do estudo foi composta por lactentes nascidos vivos em 2017 e 2018, filhos de mães que residiam em domicílios particulares situados na área urbana das 26 capitais, no Distrito Federal e nos municípios de Campinas (SP), Caruaru (PE), Imperatriz (MA), Joinville (SC), Londrina (PR), Petrópolis (RJ), Rio Grande (RS), Rio Verde (GO), Rondonópolis (MT), Sete Lagoas (MG), Sobral (CE) e Vitória da Conquista (BA).

O plano amostral do ICNV 2020^17^ organizou os setores censitários em conglomerados segundo estratos socioeconômicos. Para definir o tamanho amostral, foram considerados: efeito do desenho de 1,4; população hipotética de 1 milhão de nascidos vivos; prevalência estimada de cobertura vacinal de 70%; erro de estimativa de 5%; e intervalo de confiança de 95% – resultando numa amostra de 452 lactentes por inquérito. Em cada cidade, foram realizados de um a quatro inquéritos, dependendo do número de nascidos registrados no Sistema de Informações sobre Nascidos Vivos.^17^

### Variáveis

O estudo estimou a proporção de utilização de serviços privados de vacinação, comparou os perfis dos lactentes que utilizaram alguma vez os serviços privados com os que utilizavam exclusivamente serviços públicos e avaliou as coberturas, segundo o serviço de vacinação utilizado.

Para definir o serviço de vacinação utilizado (privado alguma vez ou público exclusivamente) e estimar o número de lactentes que utilizou alguma vez os serviços privados, considerou-se a informação dos entrevistados para a pergunta: *A criança utilizou algum serviço privado de vacinação?* Visto que diferentes composições de vacinas são utilizadas pelos serviços públicos e privados para a proteção contra as mesmas doenças, para aumentar a sensibilidade, foram também incluídos os participantes com registro, na base de dados do INCV 2020, da aplicação de vacinas ofertadas por serviços privados, que não são universalmente ofertadas pelo PNI: qualquer dose de vacina contra difteria, tétano e pertússis acelular (DTPa), vacina DTPa, *Haemophilus influenzae* b e inativada contra poliomielite (VIP) ou vacina pentavalente acelular, vacina DTPa, *Haemophilus influenzae* B, VIP e contra hepatite B ou vacina hexavalente acelular, vacina contra hepatites A e B combinadas, vacina meningocócica ACWY (MenACWY), vacina meningocócica B, segunda dose da vacina tetraviral contra sarampo, caxumba, rubéola e varicela, (SCRV) e terceira dose da vacina contra rotavírus humano. 

Outras vacinas oferecidas nos serviços privados, também oferecidas no PNI ou com formulações diferentes, foram registradas na base de dados em uma variável que combinou as diversas apresentações, sem diferenciar se a aplicação foi feita em serviços privados ou públicos, não permitindo avaliar essas vacinas. Como exemplo, a vacina SCRV, independentemente de ser aplicada em serviços públicos ou privados, foi contabilizada nas doses da vacina contra sarampo, caxumba e rubéola (SCR) ou tríplice viral, vacina contra varicela, e a vacina pneumocócica 13-valente (VPC13) foi somada à vacina pneumocócica 10-valente (VPC10).

Para se estimar a proporção de utilização de serviços privados para vacinação, foi empregada a fórmula:


$$\frac{\text{N° lactentes que utilizou alguma vez serviço privado para vacinar}}{\text{N° lactentes da amostra}}\times 100$$

Para comparar o perfil dos lactentes que utilizaram alguma vez os serviços privados de vacinação com os que utilizaram exclusivamente os serviços públicos, foram consideradas as seguintes variáveis:

características dos lactentes:
sexo (masculino e feminino);
ordem de nascimento entre os filhos (primeiro, segundo, terceiro e quarto ou mais).
características da mãe:
escolaridade (em anos: ≤ 8, 9-12, 13-15, e ≥ 16);
número de filhos vivos (um, dois, três e quatro, ou mais);
faixa etária ao nascimento do filho (em anos: ≤ 20, 20-34, ≥ 35);
situação conjugal (com ou sem companheiro);
trabalho remunerado (sim e não);
raça/cor da pele (branca, preta, parda, amarela, indígena).
características da família e do domicílio:
nível de consumo familiar (A, B, C e D – conforme a classificação da Associação Brasileira de Empresas de Pesquisa, sendo A o nível mais elevado, e D, o mais baixo);
estrato socioeconômico da área de residência (A – alto, B – médio-alto, C – médio-baixo e D – baixo); renda familiar mensal (em reais: ≤ 1.000, 1.001-3.000, 3.001-8.000 e ≥ 8.001).

Os estratos socioeconômicos da área de residência foram classificados considerando-se a renda e a alfabetização dos responsáveis pelo domicílio.^17^

A cobertura foi avaliada para cada vacina recomendada pelo PNI até os 24 meses, considerando-se a dose que representa o esquema completo ou o reforço, e para o conjunto dessas vacinas (cobertura completa). Até os 12 meses, foram consideradas: primeira dose da vacina bacilo de Calmette-Guérin (BCG) ou vacina contra tuberculose e vacina contra hepatite B; segunda dose de VPC10, vacina contra rotavírus humano e meningocócica conjugada C (MenC); e terceira dose da vacina pentavalente e VIP. Não foi incluída a vacina contra a febre amarela, pois não estava na rotina de todos os municípios incluídos no estudo no período analisado. De 12 a 24 meses, foram considerados: primeiro reforço de VPC10, MenC, vacina oral de poliovírus (VOP) e vacina contra difteria, tétano e pertússis (DTP); primeira dose de vacina contra hepatite A e vacina contra varicela; e segunda dose de SCR.

A cobertura vacinal foi calculada para vacinas aplicadas (considerando-se todas as doses aplicadas registradas na caderneta) e vacinas em dia (considerando-se apenas as doses aplicadas até 30 dias após a data prevista pelo PNI), conforme as fórmulas:


$$\text{Cobertura de vacinas aplicadas}=\frac{\text{N° lactentes com a vacina aplicada}}{\text{N° lactentes da amostra}}\times 100$$

$$\text{Cobertura de vacinas em dia}=\frac{\text{N° lactentes com a vacina aplicada em dia}}{\text{N° lactentes da amostra}}\times 100$$

$$\text{Cobertura completa de vacinas aplicadas}=\frac{\text{N° de lactentes com todas as vacinas previstas até os 24 meses aplicadas}}{\text{N° de lactentes da amostra}}\times 100$$

$$\text{Cobertura completa de vacinas em dia}=\frac{\text{N° de lactentes com todas as vacinas previstas até os 24 meses aplicadas em dia}}{\text{N° de lactentes da amostra}}\times 100$$

A proporção de utilização de serviços privados de vacinação e as coberturas completas foram descritas por:

município:
agrupados entre “interior” e “capitais”;
agrupados segundo o porte populacional (mil habitantes), a partir da classificação do Instituto Brasileiro de Geografia e Estatística (de 150 a 900 mil habitantes e > 900 mil habitantes);
regiões (Centro-Oeste, Nordeste, Norte, Sul e Sudeste).

A proporção de utilização de serviços privados de vacinação também foi descrita para cada município. A cobertura segundo vacinas foi apresentada para o conjunto dos municípios participantes do inquérito. 

### Fonte de dados

Os dados foram obtidos em entrevistas com os responsáveis pelos lactentes, bem como pela transcrição de informações sobre as vacinas aplicadas até 24 meses de fotos das cadernetas de vacinação dos lactentes.^17^

### Métodos estatísticos

As proporções de utilização de serviços privados de vacinação, as coberturas vacinais e os intervalos de confiança de 95% foram calculados no programa Stata, versão 17. O teste qui-quadrado de Pearson foi utilizado para testar diferenças estatísticas. Foram considerados estatisticamente significantes p-valores < 0,05. Para corrigir eventuais distorções na distribuição da amostra e permitir estimações sem viés, foi utilizado o módulo *survey analysi*s, considerando-se o estrato socioeconômico da área de residência, o peso calibrado e o conglomerado. Dados faltantes foram tabulados conjuntamente com as respostas “não sei”; ambos foram considerados nas análises.

### Considerações éticas

O inquérito foi aprovado pelos Comitês de Ética em Pesquisa com Seres Humanos do Instituto de Saúde Coletiva da Universidade Federal da Bahia (parecer 3.366.818, em 4/6/2019, Certificado de Apresentação de Apreciação Ética [CAAE] 4306919.5.0000.5030); e da Irmandade da Santa Casa de São Paulo (parecer 4.380.019, 4/11/2020, CAAE 39412020.0.0000.5479). O termo de consentimento livre e esclarecido foi assinado pelos responsáveis dos lactentes.^17^

## RESULTADOS

Da amostra prevista de 39.776 lactentes, foram incluídos 37.801 no inquérito. As perdas corresponderam a 6%, com variação entre os munícipios e estratos, e decorreram de recusas, impossibilidade de realização da entrevista após três tentativas e a não localização do número previsto de crianças após busca ativa. 

Entre os lactentes participantes do inquérito, 8.536 responsáveis informaram a utilização de serviços privados. A análise do registro de vacinas identificou mais 751 participantes, totalizando 9.287 (25,1%; IC_95_% 23,2;27,2) lactentes que utilizaram alguma vez os serviços privados de vacinação. 

Identificaram-se diferenças no perfil socioeconômico e demográfico das famílias, segundo serviço utilizado, exceto para o sexo do lactente. Na comparação com os lactentes que utilizaram alguma vez serviço privado com os que utilizaram somente serviço público, observou-se maior proporção de primeiros filhos (61,8% e 43,2%, respectivamente), cuja mãe possuía escolaridade ≥16 anos de estudo (75,9% e 16,8%, respectivamente), 35 anos ou mais (70% e 32,8%, respectivamente), exercendo trabalho remunerado (72,8% e 47,0%, respectivamente), e que se autodeclarou branca (69,9% e 36,2%, respectivamente). Observaram-se também maiores proporções de famílias enquadradas nos níveis A e B, considerados de consumo mais elevado (66,4% e 12,1%, respectivamente). Destaca-se, ainda, na comparação entre utilização de serviços para vacinar, a baixa proporção de lactentes que utilizaram serviços privados em mães com escolaridade ≤ 8 anos de estudo (respectivamente, 1,4% e 20,0%) e cujas famílias informaram renda mensal ≤ R$ 1.000 (respectivamente, 6,7% e 32,9%). (Tabela 1). 

**Tabela 1:** Características dos lactentes, maternas e familiares, segundo utilização de serviços privados de vacinação ^a^ em municípios brasileiros, Inquérito Nacional de Cobertura Vacinal, 2020 (n = 37.801)

Utilização de serviços privados de vacinação	Sim (9.287)			Não (28.514)			p-valor
	**n**	**%**	**IC**_95_ **%**	**n**	**%**	**IC**_95_ **%**	
Características dos lactentes							
Sexo							0,230
Masculino	4.769	49,4	46,3;52,5	14.638	51,6	49,9;53,2	
Feminino	4.518	50,6	47,5;53,7	13.876	48,4	46,8;50,1	
**Ordem de nascimento**							< 0,001
Primeiro	5.589	61,8	58,6;65,0	12.670	43,0	41,2;44,9	
Segundo	2.977	29,7	26,7;32,8	9.113	31,7	30,3;33,1	
Terceiro	553	6,5	4,9;8,5	4.155	15,0	13,9;16,1	
Quarto ou mais	157	2,0	1,2;3,1	2.553	10,2	9,0;11,5	
**Características maternas**							
**Escolaridade (anos de estudo)**							< 0,001
≤ 8	115	1,4	0,9;2,2	3.165	20,0	9,9;12,1	
9-12	231	3,4	2,4;4,9	5.263	19,6	18,0;21,2	
13-15	1.478	17,6	15,2;20,1	13.859	49,3	47,4;51,1	
≥ 16	7.311	75,9	72,7;78,6	5.326	16,8	15,3;18,4	
Não sabe/não respondeu	152	1,7	1,1;2,5	901	3,3	2,7;3,9	
**Número de filhos vivos**							< 0,001
Um	4.317	49,1	45,6;52,7	10.024	33,3	31,7;35,0	
Dois	3.965	39,2	35,7;42,8	10.062	35,7	33,9;37,4	
Três	776	9,1	7,2;11,5	5.035	17,7	16,4;19,1	
Quatro ou mais	220	2,5	1,7;3,6	3.594	10,5	9,4;11,8	
**Faixa etária ao nascimento do filho (em anos)**							< 0,001
≤ 20	30	3,6	0,2;0,6	832	3,1	2,5;3,7	
20-34	2.920	29,7	26,7;32,8	18.797	64,1	62,5;65,8	
≥35	6.302	70,0	66,8;72,9	8.729	32,8	31,1;34,4	
Não sabe/não respondeu	26	4,5	0,1;1,5	156	0,5	0,3;0,7	
**Situação conjugal**							< 0,001
Com companheiro	8.266	88,4	86,2;90,3	19.975	69,3	67,7;70,8	
Sem companheiro	856	9,7	8,0;11,6	7.543	27,3	25,8;28,8	
Não sabe/não respondeu	165	1,9	1,2;2,9	996	3,4	2,9;4,0	
**Trabalho remunerado**							< 0,001
Sim	6.882	72,8	69,7;75,8	13.564	47,0	45,4;48,6	
Não	2.259	25,7	22,8;28,7	14.161	50,2	48,6;51,7	
Não sabe/não respondeu	146	1,5	0,9;2,3	789	2,8	2,3;3,4	
**Raça/cor da pele**							< 0,001
Branca	6.365	69,9	66,3;73,3	8.862	36,2	34,0;38,5	
Preta	375	6,0	4,3;8,4	3.880	14,7	13,5;16,0	
Parda	2.252	20,5	19;23,1	14.607	44,7	42,8;46,7	
Amarela	129	2,1	1,3;3,3	239	1,2	0,7;1,9	
Indígena	10	0,0	0,0;0,1	115	0,4	0,2;0,5	
Não sabe/não respondeu	156	1,4	0,9;2,2	811	0,3	2,2;3,3	
**Características familiares**							
**Estrato socioeconômico da área de residência**							< 0,001
A	3.642	27,2	23,0;31,9	4.691	6,7	5,6;7,9	
B	2.897	22,0	18,4;26,0	6.521	8,9	7,7;10,3	
C	2.003	25,8	21,9;30,0	7.989	19,17	17,3;21,1	
D	745	24,9	20,8;29,5	9.313	65,2	62,3;68,0	
**Nível de consumo familiar**							< 0,001
A	1.645	14,4	11,9;17,4	290	0,8	0,6;1,2	
B	5.135	52,0	48,0;55,7	3.866	11,3	10,0;12,6	
C	1.747	22,4	19,3;25,7	9.868	37,6	35,6;39,6	
D	488	7,8	6,1;9,9	13.520	46,8	44,7;49,0	
Não sabe/não respondeu	272	3,4	2,4;4,9	970	3,5	2,9;4,1	
**Renda familiar mensal (em reais)**							< 0,001
≤ 1.000	359	6,7	5,1;8,6	8.327	32,9	30,8;35,1	
1.000-3.000	1.115	13,6	11,1;16,6	11.551	40,9	38,8;43,1	
3.000-8.000	2.718	25,2	22,0;28,7	4.741	13,0	11,7;14,4	
≥ 8.001	3.646	29,6	26,1;33,4	974	2,3	1,9;2,9	
Não sabe/não respondeu	1.449	24,8	20,8;29,4	2.921	10,7	10,0;12,6	

a) Sim: participantes que utilizaram algum vez serviços privados de vacinação; Não: participantes que utilizaram exclusivamente serviços públicos de vacinação.

Foram observadas diferenças territoriais na utilização de serviços privados de vacinação, que variou de 3,9% (IC_95_% 1,2;7,6), em Rio Branco, a 58,4% (IC_95_% 42,1;73,1), em Vitória (Figura 1), tendo sido maior nas capitais (p = 0,004) e nos municípios de porte 5 (p < 0,001). Proporções maiores que 20% foram observadas em 14 das 26 capitais, em Brasília, e em três cidades do interior do Sudeste, e superaram 50% em Vitória e Florianópolis. Observou-se maior utilização de serviços privados de vacinação nas regiões Sul e Sudeste (respectivamente, 33,2% IC_95_% 27,8;39,1 e 30,5% IC_95_% 26,6;34,8), e menor nas regiões Norte e Nordeste (respectivamente, 13,5% IC_95_% 9,9;18,3 e 19,2% IC_95_% 15,8;23,0) (p < 0,001) (Figura 2). 

**Figura 1:** Utilização de serviços privados de vacinação^a^ por municípios, Inquérito Nacional de Cobertura Vacinal, 2020 (n = 37.801)

**Figura 2:** Utilização de serviços privados de vacinação^a^ segundo regiões brasileiras, categorização (“interior” e “capital”) e porte populacional do município, Inquérito Nacional de Cobertura Vacinal, 2020 (n = 37.801)

Considerando-se o conjunto de municípios, não foram encontradas diferenças na cobertura completa até 24 meses entre lactentes que utilizaram exclusivamente os serviços públicos ou alguma vez o privado. A cobertura completa segundo vacinas aplicadas foi, respectivamente, de 60,3% e 59,5% (IC_95_% 58,6;62,0, IC_95_% 55,9;63,0, e p = 0,704), e, segundo vacinas em dia, de 10,3% e 9,4% (IC_95_% 9,1;11,6, IC_95_% 7,4;11,8, e p = 0,473). Observaram-se diferenças significativas (p = 0,034) somente na região Norte para vacinas em dia, com cobertura de 3,0% (IC_95_% 2,3;4,1) para o serviço público e de 0,7% (IC_95_% 0,2;3,0) para o privado (Tabela 2). 

**Tabela 2:** Cobertura vacinal de vacinas aplicadas e em dia para o conjunto de vacinas recomendadas até 24 meses, segundo utilização de serviços privados de saúde, ^a^ de acordo com as características dos municípios e regiões do país, Inquérito Nacional de Cobertura Vacinal (n = 37.801)

**Cobertura vacinal**	**Vacinas aplicadas**			**Vacinas em dia**		
	**Privado (%)**	**Público (%)**	**p-valor**	**Privado (%)**	**Público (%)**	**p-valor**
**Brasil**						
Total	59,5	60,3	0,704	9,4	10,3	0,473
**Tipo do município**						
Interior	64,6	61,7	0,538	10,6	10,9	0,885
Capital	59,2	60,1	0,656	9,3	10,2	0,492
**Porte do município (mil habitantes)**						
150-900	54,6	57,8	0,211	7,1	6,5	0,633
> 900	60,8	61,3	0,859	10,0	11,8	0,274
**Regiões**						
Norte	53,8	55,7	0,762	0,7	3,0	0,034
Nordeste	50,7	58,6	0,122	5,8	9,0	0,098
Centro-Oeste	61,3	63,5	0,539	10,0	10,7	0,694
Sudeste	61,1	60,3	0,824	11,6	13,6	0,410
Sul	67,1	68,4	0,753	9,0	10,0	0,675

a) Privado: participantes que utilizaram alguma vez serviços privados de vacinação; Público: participantes que utilizaram exclusivamente serviços públicos de vacinação.

Ao se avaliar a cobertura segundo vacinas aplicadas, observa-se maior cobertura da primeira dose de varicela entre lactentes que utilizaram os serviços privados. Entre as vacinas indicadas até 12 meses, a cobertura em dia da segunda dose de VPC10, vacina contra rotavírus humano e MenC e da terceira dose da vacina pentavalente e VIP foi mais elevada entre lactentes que utilizaram os serviços privados. Para as vacinas indicadas entre 12 e 24 meses, a cobertura em dia do primeiro reforço de VOP e da segunda dose de SCR foi mais elevada entre lactentes que utilizaram os serviços públicos; por outro lado, a cobertura em dia da primeira dose de SCR, vacinas contra hepatite A e varicela e do primeiro reforço de VPC10 foi mais elevada entre lactentes que utilizaram serviços privados (Tabela 3).

**Tabela 3:** Cobertura vacinal para vacinas aplicadas e em dia, por vacinas recomendadas até 12 meses e de 12 a 24 meses, segundo tipo de serviço utilizado para vacinação, ^a^ Inquérito Nacional de Cobertura Vacinal 2020 (n = 37.801)

**Vacina**	**Vacinas aplicadas**			**Vacinas em dia**		
	**Publico**	**Privado**	**p-valor**	**Público**	**Privado**	**p-valor**
	**% (IC_95_ % )**	**% (IC_95_ % )**		**% (IC_95_% )**	**% (IC_95_ % )**	
**Até 12 meses**						
Primeira dose da vacina contra tuberculose	89,6 (87,3;91,5)	89,8 (88,5;91,0)	0,839	82,3 (79,3;85,0)	84 (82,6;85,4)	0,293
Primeira dose da vacina contra hepatite B	89,3 (87,0;91,2)	88,6 (87,2;89,8)	0,537	86 (83,5;88,3)	85,5 (84,1;86,9)	0,756
Segunda dose da vacina pneumocócica 10	91,1 (89,0;92,8)	90,2 (89,0;91,3)	0,407	78,7 (78,8;81,3)	66,3 (64,4;68,0)	< 0,001
Segunda dose da vacina contra rotavírus humano	83,6 (80,7;86,1)	81,7 (80,3;83,1)	0,248	72,8 (69,2;76,0)	64,5 (62,7;66,4)	< 0,001
Segunda dose da vacina meningocócica C	89,8 (87,7;91,5)	89,3 (88,1;90,4)	0,663	70,9 (67,8;73,8)	58,7 (56,7;60,7)	< 0,001
Terceira dose da vacina pentavalente	86,7 (84,0;89,0)	88,6 (87,3;89,8)	0,148	66,1 (62,8;69,3)	53 (50,8;55,2)	< 0,001
Terceira dose da vacina inativada contra poliomielite	86,3 (83,6;88,6)	88,6 (87,3;89,8)	0,084	66,8 (63,3;70,0)	55,5 (53,5;57,5)	< 0,001
**12-24 meses**						
Primeiro reforço da vacina pneumocócica 10	86,1 (83,4;88,4)	84,7 (83,3;85,9)	0,309	65,7 (62,6;68,7)	51,5 (49,7;53,3)	< 0,001
Primeiro reforço da vacina meningocócica C	84,0 (81,4;86,3)	81,6 (80,2;82,8)	0,108	48,0 (45,1;51,3)	47,4 (45,5;49,3)	0,229
Primeira dose da vacina contra hepatite A	89,6 (87,4;91,4)	87,7 (86,4;88,9)	0,112	56,5 (53,3;59,7)	49,3 (47,7;50,9)	< 0,001
Segunda dose da vacina tríplice viral contra sarampo, rubéola e caxumba	84,3 (81,2;87,0)	81,5 (80,1;82,9)	0,103	39,8 (36,3;43,4)	42,7 (41,0;44,0)	0,019
Primeiro reforço da vacina oral de poliovírus	84,4 (81,8;86,8)	86,9 (85,6;87,9)	0,078	39,5 (36,5;42,6)	44,3 (42,5;46,1)	0,039
Primeiro reforço da vacina tríplice bacteriana contra difteria, tétano e pertússis	82,9 (80,2;85,3)	84,7 (83,4;85,9)	0,218	37,1(33,9;40,4)	37,2 (35,4;39,0)	0,427
Primeira dose da vacina contra varicela	89,3 (87,1;91,1)	86,3 (84,9;87,6)	0,019	57,2 (54,0;60,3)	44,1 (42,4;45,9)	< 0,001

a) Privado: participantes que utilizaram alguma vez serviços privados de vacinação; Público: participantes que utilizaram exclusivamente serviços públicos de vacinação.

## DISCUSSÃO

A presente contribuição trata de tema pouco abordado, que possibilitou caracterizar a utilização de serviços privados na vacinação de lactentes no país. Um em cada quatro lactentes participantes do INCV 2020 utilizou alguma vez serviços privados para vacinação, com maiores proporções em capitais, cidades de maior porte e regiões Sul e Sudeste. Foram evidenciadas baixas coberturas completas até 24 meses, especialmente para vacinas aplicadas em dia, sem diferenças estatisticamente significativas entre lactentes que utilizaram serviços públicos e privados de vacinação. 

Comparando-se os dados, referentes às capitais, do atual inquérito com os do realizado em 2007-2008, evidenciou-se aumento de 60% na utilização de serviços privados de vacinação^18^ e incremento no número de capitais em que a utilização de serviços privados superou 20%, passando de três capitais para 15.^20^ O aumento da participação dos serviços privados na imunização de crianças também foi observado em outros países, como Índia e Sri Lanka.^8,9^

As diferenças demográficas na utilização de serviços privados de vacinação possivelmente estão associadas ao grau de desenvolvimento socioeconômico local e à maior presença de serviços privados no território, facilitando o acesso da população a esses serviços. Vale ressaltar que o INCV 2020 incluiu somente capitais e municípios do interior com mais de 100 mil habitantes, o que pode explicar em parte as elevadas proporções de utilização de serviços privados de vacinação observadas. Nessa mesma linha, estudos prévios estimaram em 37% a utilização desses serviços na capital do Sri Lanka,^9^ e em 35%, da Argentina,^14^ contrastando com os 9% encontrados por estudo realizado em municípios com mais de 20 mil habitantes de todas as regiões argentinas.^21^

São fatores que contribuem para a participação expressiva dos serviços privados na vacinação de lactentes: o lançamento de novas vacinas não incorporadas imediatamente ao PNI; o desabastecimento de alguns imunobiológicos nas salas de vacinação do Sistema Único de Saúde (SUS);^22^ a conveniência no atendimento de serviços privados, incluindo-se horários ampliados,^10,13^ proximidade da residência em áreas de maior estrato socioeconômico^10^ e, mais recentemente, aplicação de vacinas em farmácias.^5^

No Brasil, os serviços público e privado de saúde coexistem, compartilhando usuários.^23^ Posto que a imunização não faz parte dos procedimentos obrigatórios definidos pela Agência Nacional de Saúde Suplementar, em geral os planos de saúde privados não cobrem vacinas, e seus segurados utilizam rotineiramente o serviço público para vacinação^24^ ou pagam diretamente por vacinas no serviço privado. Maiores proporções de utilização de serviços de vacinação privados por crianças cujos pais possuíam planos de saúde foram descritas,^21^ o que pode indicar que planos de saúde facilitam o acesso a vacinas não oferecidas universalmente no PNI.

Com relação ao perfil socioeconômico, a maior utilização de serviços privados de vacinação por lactentes cuja família possuía maior nível de consumo e renda familiar, e cuja mãe exercia trabalho remunerado, aponta a disponibilidade financeira como um fator importante no acesso a esses serviços. O estrato socioeconômico, a renda e a classe social foram associados à maior utilização de serviços privados na Índia e no Sri Lanka.^8,9^

Se, por um lado, o maior uso do serviço privado por lactentes cujas mães possuíam alta escolaridade pode indicar maior conhecimento sobre vacinas disponíveis em serviços privados, por outro pode representar um fator de confusão para variáveis como a renda. As mães possuírem companheiro é outra característica que pode gerar confusão quanto à renda. Analogamente ao observado em nossos resultados, nos Estados Unidos, filhos de pais que não possuíam ensino superior e que eram solteiros utilizavam mais serviços públicos de vacinação.^16^ Por outro lado, um estudo que avaliou gastos com vacinas na população adulta com diabetes, em São Paulo, não identificou diferenças na utilização do serviço público segundo renda, escolaridade e situação conjugal.^25^

A ordem de nascimento do participante e o número de filhos vivos influenciaram no uso de serviços privados de vacinação, sendo maior para os primogênitos. Tais achados também foram encontrados no Sri Lanka.^9^ A relação inversa do número de filhos e do uso de serviços privados, sobretudo a partir do terceiro filho, pode indicar migração para o uso de serviços públicos, devido à pressão econômica. 

Considerando-se a avaliação da cobertura completa até 24 meses, lactentes que utilizaram serviços públicos e privados de vacinação apresentaram coberturas vacinais semelhantes. Corroborando nossos resultados, Agampodi et al. não encontraram diferenças na cobertura segundo tipo de serviço.^9^ Diferindo do encontrado, o inquérito de 2007-2008 apontou maior probabilidade de serem completamente vacinados aos 18 meses os lactentes que foram vacinados exclusivamente em serviços públicos.^18^ Em uma tendência oposta, estudos realizados na Argentina e nos Estados Unidos associaram a completude vacinal ao uso exclusivo de serviços privados de vacinação e ao uso concomitante de ambos os serviços.^14,15^ Além disso, dados de inquérito nacional de imunização nos Estados Unidos, que avaliou a cobertura aos 24 meses, apontou maior cobertura entre as crianças que possuem seguro de saúde privado.^26^

 Na avaliação segundo vacinas aplicadas, a cobertura para a primeira dose de varicela foi mais elevada entre lactentes que utilizaram serviços privados. Uma possível justificativa para essa situação é a diferença na indicação dessa vacina pelo calendário adotado nos serviços, sendo ela indicada no privado aos 12 meses, e no público, aos 15 meses.^2,7^

Para mais de 50% das vacinas avaliadas, foram observadas maiores coberturas em dia entre lactentes que utilizaram serviços privados. Contudo, ao se avaliar a cobertura completa, não foram identificadas diferenças segundo tipo de serviço de vacinação utilizado, com coberturas muito baixas em ambos. Estudo em Singapura mostra dados similares quanto ao atraso no recebimento de uma ou mais doses de vacina, porém divergentes quanto à análise do serviço utilizado, sendo o atraso vacinal mais frequente em crianças vacinadas no setor privado ou em múltiplos provedores.^13^ Nessa mesma linha, Simpson et al. identificaram melhores proporções de vacinação em dia entre as crianças que se vacinavam em serviços públicos em comparação às que se vacinavam em serviços privados.^16^

Como limitações deste estudo, deve-se citar a possibilidade de viés na classificação dos participantes quanto ao uso dos serviços privados. A informação dos responsáveis quanto à utilização de serviço privado para vacinar pode estar incorreta, pois nem sempre quem respondeu ao questionário foi o familiar que levava a criança para vacinar. Outra limitação foi o fato de não ter sido transcrito, das cadernetas de vacinas para a base de dados, o serviço utilizado para cada dose de vacina, impossibilitando identificar todas as doses aplicadas por serviços privados, principalmente para vacinas que estão disponíveis no PNI, o que pode ter subestimado a utilização desse serviço. Tal situação também não permitiu identificar lactentes que receberam todas as doses nos serviços privados. A fim de identificar o maior número possível de lactentes que utilizaram alguma vez os serviços privados, foram incluídos participantes com registro, na base de dados, da aplicação de algumas vacinas ofertadas por esses serviços, não disponíveis universalmente no PNI. Contudo, salienta-se que essas vacinas poderiam ter sido aplicadas nos CRIE,^6^ o que poderia, por outro lado, superestimar a utilização de serviços privados. 

A frequência de utilização dos serviços privados observada aponta a importância da articulação entre os serviços públicos e privados de vacinação, para garantir a qualidade na administração de vacinas e o correto registro das doses aplicadas no sistema de informações do PNI.^8,12,27^ Apesar de ser obrigatório o registro de dados individuais referentes às vacinas aplicadas por serviços privados no sistema de informação,^28,29^ não existem estratégias nacionais de monitoramento dos serviços privados. 

O acesso exclusivo a algumas vacinas por aqueles que podem utilizar serviços privados de vacinação contrapõe os princípios de equidade e universalidade do SUS.^4,30^ Todavia, observa-se que o PNI, com uma estrutura de atendimento descentralizada, em toda a rede de atenção primária do SUS, tem importante contribuição na redução das desigualdades sociais e regionais, viabilizando o acesso à vacinação para todos os brasileiros, em todas as localidades.^1^ A semelhança das coberturas completas segundo vacinas aplicadas e vacinas em, dia entre lactentes que utilizam serviços públicos e privados, reforça a capilaridade do PNI no território brasileiro. 

As metas de cobertura vacinal não alcançadas e a vacinação fora do período recomendado, independentemente do serviço utilizado, apontam a necessidade de implementar ações para enfrentamento dessa situação. O monitoramento detalhado da situação vacinal, incluindo a avaliação do registro de doses aplicadas pelos serviços privados no sistema de informação do PNI, e a compreensão dos fatores associados à incompletude e ao atraso vacinal, podem orientar a adoção de novas estratégias visando restabelecer altas coberturas e garantir a vacinação em dia.
